# Anti-Swelling Aramid-Nanofiber-Reinforced Zwitterionic Polymer Hydrogel for Strain Sensors

**DOI:** 10.3390/ma18081800

**Published:** 2025-04-15

**Authors:** Zeyu Chen, Wenbin Zhong

**Affiliations:** College of Materials Science and Engineering, Hunan University, Changsha 410082, China; zychen@hnu.edu.cn

**Keywords:** aramid nanofibers, zwitterionic polymer, hydrogel, strain sensor

## Abstract

Zwitterionic polymer hydrogels have great application prospects in wearable electronic devices due to their antifouling and excellent biocompatibility. However, its strong hydrophilicity often leads to easy swelling and poor mechanical properties. In this study, aramid nanofiber (ANF)-reinforced zwitterionic ion hydrogels were synthesized by the one-step free radical polymerization of *N*-acryloyl glycinamide (NAGA), *N*-[Tris (hydroxymethyl) methyl] acrylamide (THMA) and sulfobetaine methacrylate (SBMA) monomers in the presence of ANFs. A large number of hydrogen bonds were formed between the amide groups of the ANFs and the amide groups of the NAGA units/the hydroxyl groups of the THMA units/the sulfonic groups of the SBMA units, which improved the internal interface force of the hydrogel. The obtained ANF-reinforced hydrogel had an anti-swelling property, and its swelling ratio and tensile strength were 25% and 170% of those of the hydrogel without the addition of ANFs. By introducing lithium chloride as an electrolyte to improve its ion conductivity and subsequently assembling it into strain sensors, it exhibited a high sensitivity (GF = 1.12), short response and recovery times (100 ms and 150 ms), and excellent cycling stability. This work provides a feasible strategy for anti-swelling wearable strain sensors.

## 1. Introduction

With the rapid development of technology, intelligent devices are playing an important role in various aspects of people’s lives. Among numerous intelligent devices, flexible electronic devices have strong flexibility and can adapt to various environments to meet people’s needs, which has attracted much attention, especially in fields such as human–machine interfacing [1,2], biomedicine [3], electronic skin [4,5], energy acquisition [6] and health monitoring [7,8]. Flexible sensors can convert an external response (force, light, temperature, etc.) into measurable electrical signals, and their simple structure, manufacturing process and low cost have become one of the recent research hotspots [9,10,11,12]. Hydrogels are usually formed by the physical or chemical crosslinking of hydrophilic natural polymers or synthetic polymers, and the conductivity, elasticity, mechanical strength and other characteristics can be adjusted through structural design [13,14]. These excellent features make them one of the ideal candidates for flexible sensors.

Traditional polymer hydrogels used as flexible sensors are mainly divided into conductive hydrogels containing conductive materials in the structural network and ionic conductive hydrogels composed of polymers and inorganic salts/ionic liquids [15,16]. Ionic conductive hydrogels mainly include polyacrylamide [17], polyvinyl alcohol [18], polyacrylic acid [19] and zwitterionic polymers (such as sulfobetaine methacrylate (SBMA) [20] and 2-Methacryloyloxyethyl phosphorylcholine (MPC) [21]). Among them, zwitterionic monomers, also known as internal salts, are small molecules containing cations and anions on the same unit. Due to their low pollution and excellent biocompatibility, they can be used as biocompatible materials. The interaction between zwitterionic polymers and salt ions may affect their ionic conductivity. For example, the electrostatic interaction between SBMA and salt ions may affect the ion conductivity through ion-hopping mechanisms [22], while the ice-like inclusion structure formed by electrostatic induction between MPC and water molecules affects the hydration state of ions, which exhibits at least 130% of the ion conductivity of the corresponding inorganic salt solution [21]. These fascinating properties have led to extensive research on zwitterionic polymers in the field of flexible electronics. However, the mechanical strength of hydrogels based on zwitterionic polymers is usually poor due to their good hydrophilicity. Researchers improved the mechanical properties of zwitterionic hydrogels to some extent by polymerizing zwitterionic monomers with other monomers [20,23,24,25]. For example, the compression strength of a zwitterionic polymer hydrogel prepared with *N*-(2-hydroxyethyl)acrylamide (HEAA) and SBMA can reach ~20 kPa, compared with PSBMA, which cannot form a hydrogel [20]. The tensile strength of a zwitterionic ion hydrogel formed by the copolymerization of acrylic acid (AA)/*N*-[Tris (hydroxymethyl) methyl] acrylamide (THMA)/SBMA can reach ~12 kPa [23]. In conclusion, it is feasible to improve the mechanical properties of zwitterionic hydrogels by introducing other monomers.

Hydrogels are mainly formed by chemical crosslinking (chemical bonds) and physical crosslinking (hydrogen bonds, electrostatic adsorption, etc.). When they are immersed in water, the weak interaction forces in the polymer (such as hydrogen bonds between hydroxyl groups and electrostatic adsorption between zwitterions) are disrupted by the hydration of their hydrophilic units [26,27]. In practical applications, most hydrogels will expand to absorb a large amount of water when working in a water-rich environment (such as soaking inorganic salts/ionic liquids to obtain higher conductivity or injecting them into tissues) [28,29]. At this time, the mechanical properties of the hydrogel will decline sharply after swelling, which seriously affects its practical application [26,27,28,29]. Huang et al. utilized surface packaging technology with poly(styrene-butadiene-styrene) as an elastic hydrophobic skin to chemically encapsulate a series of polyamide hydrogels with water retention and anti-swelling [30]. This method isolated the solvent exchange between the hydrogel and the outside world, making the hydrogel unable to soak in inorganic salts/ionic liquids to obtain a higher conductivity. Xu et al. introduced octadecyl methacrylate as a hydrophobic group in the acrylic acid and SBMA system, which adjusted the hydrophobic and hydrophilic interaction of the hydrogel and reduced the swelling ratio of the hydrogel by nearly half [31]. When the concentration of the *N*-acryloyl glycinamide (NAGA) monomer exceeds 10 wt.%, PNAGA hydrogel has a high strength and non-swelling property [32], which is attributed to the large number of hydrogen bonds between the amide groups in its polymer chains. The bond energy of these hydrogen bonds is strong, which can prevent the hydrogel from swelling. When SBMA is copolymerized with NAGA (the mass ratio of SBMA/NAGA is 2:1 and a small amount of 1-carboxy-*N*-methyl-*N*-di(2-methacryloyloxy-ethyl)methanaminium inner salt (CBMAX) as a chemical crosslinking agent), the copolymer exhibits a high swelling rate (over 3000%) [33]. However, when glycerol is introduced into the system, its swelling rate decreases and its elongation at break increases. This means that introducing monomers with hydroxyl (such as THMA) into the system may be beneficial to reducing the swelling rate of hydrogels and improving the elongation at break. When NAGA and THMA are copolymerized to form a hydrogel, the hydrogel will still swell, which may be attributed to the fact that the hydrogen bond energy formed between the amide groups/hydroxyl groups and hydroxyl groups/amide groups is lower than that formed between the amide groups, and these hydrogen bonds with a low bond energy are partially broken during the swelling process [34]. In order to further reduce the swelling rate of a NAGA-based hydrogel, it is necessary to increase the strong hydrogen bond in the NAGA-based copolymer system.

Deprotonated aramid nanofibers (ANFs), which are deprotonated from poly(p-phenylene terephthalamide) (PPTA) fibers in KOH/DMSO solution [35], overcome the difficult process ability of PPTA fibers while retaining the high mechanical properties and thermal stability of PPTA. ANFs are rich in amide groups and easily form strong hydrogen bonds with the amide groups of NAGA, thus enhancing the mechanical properties of NAGA-based hydrogels and reducing their swelling ratio. In the present work, a new PNTS zwitterionic hydrogel was prepared by one-step radical polymerization of NAGA, THMA and SBMA, and its mechanical properties before and after swelling were investigated. By introducing aramid nanofibers (ANFs) to increase the hydrogen bond crosslinking between the amide groups in the PNTS zwitterionic hydrogel, an ANF-reinforced zwitterionic hydrogel a-PNTS with a low swelling ratio and high mechanical strength was obtained. In addition, lithium chloride was introduced into the PNTS and a-PNTS hydrogels to form PNTS-LiCl and a-PNTS-LiCl hydrogels. It was found that the introduction of ANFs slightly reduced the overall ionic conductivity of the hydrogel. By assembling the PNTS LiCl and a-PNTS LiCl hydrogels into strain sensors, the application prospects in the field of wearable strain sensors were studied.

## 2. Experimental Section

### 2.1. Materials

Poly(p-phenylene terephthalamide) (PPTA) fibers (Kevlar 1000D, the diameter of a single fiber was about 12 µm) were obtained from Dongguan Sovetl Co., Ltd., Dongguan, China. Dimethyl sulfoxide (DMSO), Potassium hydroxide (KOH), ammonium persulfate (APS), lithium chloride (LiCl) and acetone ethanol were all AR grade and purchased from Sinopharm Chemical Reagent Co., Ltd., Shanghai, China. Sulfobetaine methacrylate (SBMA), *N*-(2-amino-2-oxyethyl)acrylamide (NAGA) and *N*-[Tris (hydroxymethyl) methyl] acrylamide (THMA) were purchased from Meryer Chemical Technology Co., Ltd. (Shanghai, China). All the above chemicals were used as received without further purification.

### 2.2. Preparation of Aramid Nanofiber (ANF) Dispersion

The deprotonated aramid nanofiber (ANF) dispersion (1.0 wt.%) was prepared according to the literature: 1.5 g of KOH and 1 g of PPTA fiber was successively added into 100 mL DMSO under stirring. The mixture was continuously stirred at 30 °C for 7 days to prepare a dark red ANF dispersion [35]. The ANFs were isolated from the solution according to the literature to obtain ANF powders by diluting the above ANF dispersion with DMSO to 0.1 wt.% and then adding an equal volume of deionized water under stirring for 12 h. The ANFs were collected by filtration and washed three times with deionized water. Finally, the ANFs were collected with acetone and dried in a vacuum at room temperature to obtain the ANF powder [36].

### 2.3. Preparation of a Series of a-PNTS, PNTS, PNT and PNS Hydrogels

All the hydrogels were synthesized through the following process: the ANF powder was added into 2 mL deionized water under vigorous stirring (1200 rpm) for 24 h and then an ultrasonic treatment for 2 h. Subsequently, NAGA (400 mg), THMA and SBMA (total mass of THMA and SBMA was 200 mg) were added to the above mixture under stirring for 1 h and then underwent an ultrasonic treatment for 10 min. An APS (3 mg dissolved in 60 μL deionized water) aqueous solution was added to the above mixture under stirring, and then the mixture was transferred to a specific apparatus. The a-PNTS hydrogel was obtained by reacting the mixture at 70 °C for 3 h. The specific ratio and hydrogel naming can be found in Table 1. All the obtained hydrogels were immersed in deionized water for 30 min to remove unreacted monomers and residual products. The preparations of all hydrogels were repeated 5 times for subsequent testing.

### 2.4. Material Characterization

Scanning electron microscopy (SEM) images of the samples were measured using a Hitachi S-4800 (Hitachi, Tokyo, Japan). X-ray photoelectron spectroscopy (XPS) measurements were carried out on a Thermo ESCALAB 250Xi (Thermo Fisher Scientific, Waltham, MA, USA). Fourier transform infrared (FTIR) spectra of the samples were characterized on a Thermo iS50 spectrometer (Thermo Fisher Scientific, Waltham, MA, USA). The electrochemical performances of the as-prepared samples were carried out using an electrochemical workstation (CHI760E, Shanghai CH Instruments Co., Ltd., Shanghai, China). The pressure sensor device was constructed by a sandwiched PNTS-based hydrogel and two pieces of Au as a collector and then wrapped with polyethylene film. The sensitivity of the sensor was calculated using the following equations:

(1) $$∆I=\frac{I-I_{0}}{I_{0}}$$

(2) $$S=\frac{∆I-∆I_{0}}{P-P_{0}}$$

where I is the real-time current, I_0_ is the current in the initial state, P is the real-time pressure, and P_0_ is the pressure in the initial state.

The relative resistance change and gauge factor (GF) were obtained using the following equations:

(3) $$∆R=\frac{R-R_{0}}{R_{0}}$$

(4) $$S=\frac{∆R-∆R_{0}}{\varepsilon -\varepsilon_{0}}$$

where R is the real-time current, R_0_ is the current in the initial state, ε is the strain, and ε_0_ is the strain in the initial state.

### 2.5. Measurement of Mechanical Properties

The mechanical properties of all the as-prepared samples were measured with an electronic dynamometer (SC-XD-50, SHENCE Intelligent Co., Ltd., Shenzhen, China) at room temperature. The tensile samples were prepared in a dumbbell mold (15 mm in total length, 2 mm in inner width and 5 mm in gauge length) and stretched at a speed of 5 mm min^−1^. The compressed samples were prepared in a cylindrical mold with a diameter of 10 mm and a height of 10 mm and compressed at a speed of 2 mm min^−1^. The mechanical properties of each sample were repeatedly tested with at least three sets in parallel to obtain a mean value.

### 2.6. Measurement of Swelling Ratio

The PNT, PNS, PNTS and a-PNTS hydrogels were soaked in water at room temperature, and their swelling ratios were recorded through their mass changes. The swelling ratio (SR) of each sample was repeatedly tested using at least three sets in parallel to obtain a mean value, and it was calculated according to the formula

(5) $$SR=\frac{W-W_{0}}{W_{0}}$$

where W is the mass of the hydrogel after soaking in the solution and W_0_ is the initial mass of the hydrogel.

### 2.7. Measurement of Ionic Conductivity

The electrochemical performances were studied on a CHI760E electrochemical working station, where two Au sheets were connected to the top and bottom of the samples to form a sandwich structure. The solution resistance of the sample was measured using the Electrochemical Impedance Spectroscopy (EIS) method. The fully swollen PNTS and a-PNTS hydrogels were immersed in a LiCl solution with different concentrations (1–8 M) at room temperature for 24 h, and then their ionic conductivities were measured. The ionic conductivity of each sample was repeatedly tested with at least three sets in parallel to obtain a mean value. The ionic conductivity (σ) was calculated according to the formula

(6) $$\sigma =\frac{L}{R\times S}$$

where R is the resistance, S is the cross-sectional area of the measured electrolyte and L is the thickness of the measured sample.

## 3. Results and Discussion

**Figure 1:**
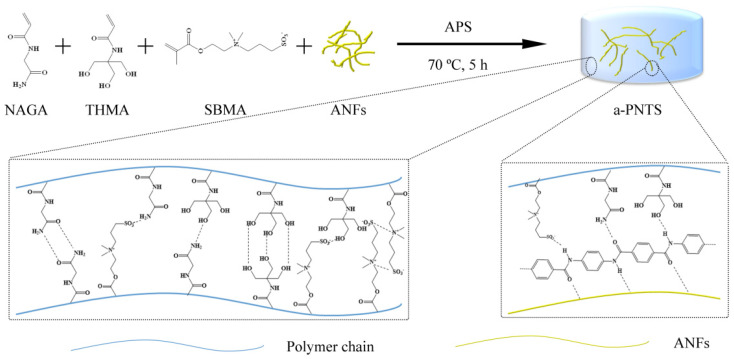
Schematic illustration of the fabrication of the a-PNTS hydrogels.

The a-PNTS hydrogel was prepared by one-step radical polymerization. The schematic illustration of the preparation process is shown in Figure 1. The aramid nanofibers (ANFs) were dispersed in water, and then *N*-(2-amino-2-oxyethyl)acrylamide (NAGA), *N*-[Tris (hydroxymethyl) methyl] acrylamide (THMA) and sulfobetaine methacrylate (SBMA) monomers were added in turn. Hydrogen bonds were formed between the amide groups of the ANFs through amide groups of the NAGA, hydroxyl groups of THMA and sulfonic acid groups of SBMA, which made the NAGA, THMA and SBMA monomers partially attach to the surfaces of ANFs. After mixing, ammonium persulfate solution was added and heated at 70 °C for 5 h to obtain an a-PNTS hydrogel. The PNTS, PNT and PNS hydrogels were prepared by the above method without adding ANFs, with ANFs and SBMA, and with ANFs and THMA, respectively. The crosslinked network formed by NAGA endowed the hydrogel with excellent mechanical strength. The ionic conductive network of the hydrogel was constructed by the interaction between the SBMA and the Li^+^ and Cl^−^. The ductility of the hydrogel was improved by the introduction of THMA. These hydrogels were mainly combined by chemical crosslinking and physical crosslinking, mainly including covalent bonds formed during monomer polymerization, hydrogen bonds formed between the amide groups at the ends of NAGA units, hydrogen bonds formed between the amide groups of ANFs and the amide groups at the ends of NAGA units, hydrogen bonds formed between the hydroxyl groups at the ends of THMA units, hydrogen bonds formed between the amide groups at the ends of NAGA units and the hydroxyl groups at the ends of THMA units, hydrogen bonds formed between the amide groups of ANFs and the hydroxyl groups at the ends of THMA units, hydrogen bonds formed between the sulfonic acid groups at the ends of SBMA units and other units, and electrostatic adsorption between SBMA units. In these crosslinks, when the hydrogel was immersed in water, the covalent bond formed during the monomer polymerization and the hydrogen bond formed between amide groups did not break because of its strong bond energy. For the PNTS, PNT and PNS hydrogels, the hydrogen bonding between the THMA/SBMA units and the NAGA units reduced the number of hydrogen bonds formed between the amide groups at the ends of the NAGA units, so they had a certain swelling property. However, the introduction of ANFs increased the number of hydrogen bonds formed between the amide groups in the system, and the swelling degree of the a-PNTS hydrogel was lower than that of the PNTS hydrogel.

### 3.1. Chemical Composition of Hydrogels

**Figure 2:**
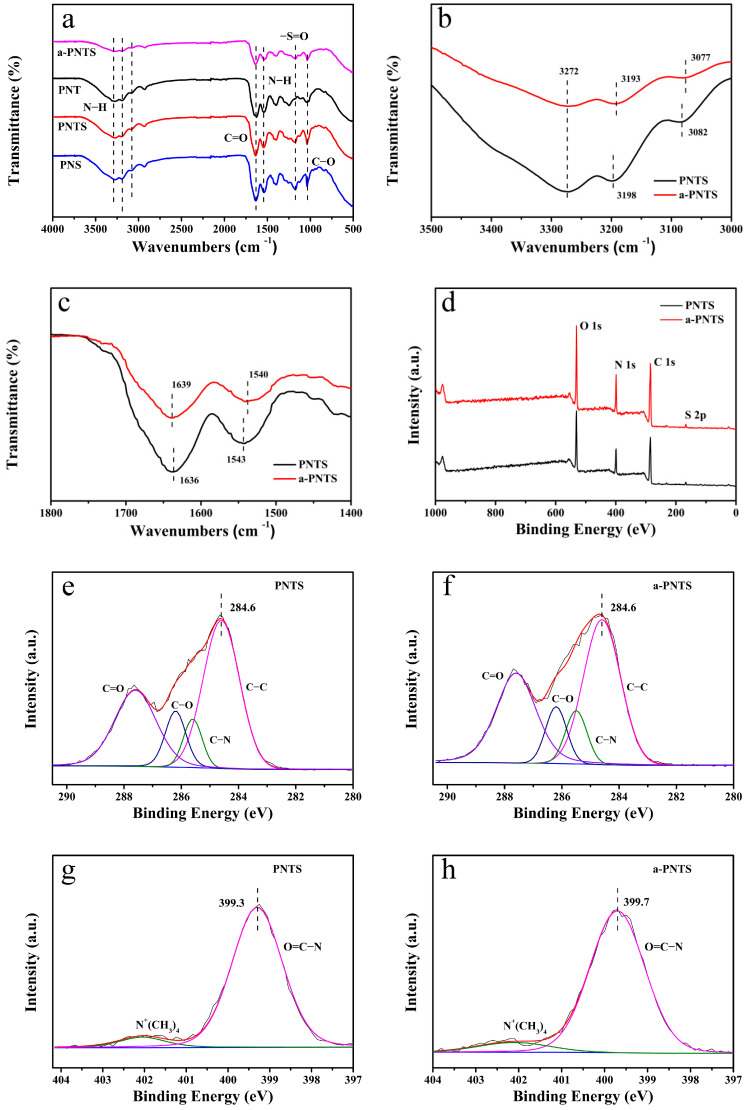
(**a**) FTIR spectra of a-PNTS, PNTS, PNT and PNS. (**b**,**c**) Comparison of partial FTIR spectra comparison of a-PNTS and PNTS. (**d**) XPS spectra of PNTS and a-PNTS. Decomposed C 1s spectra of (**e**) PNTS and (**f**) a-PNTS. Decomposed N 1s spectra of (**g**) PNTS and (**h**) a-PNTS.

The intermolecular interactions in the PNTS, PNS, PNT and a-PNTS hydrogels were analyzed by Fourier-transform infrared (FTIR) spectroscopy (Figure 2a). The characteristic peaks at approximately 3272 cm^−1^, 3198 cm^−1^ and 3082 cm^−1^ were assigned to the stretching vibrations of N−H; the peak at 1543 cm^−1^ was assigned to the bending vibrations of N−H; and the peaks at 1636 cm^−1^ and 1038 cm^−1^ were assigned to the stretching vibrations of C=O and C−O, respectively. The characteristic peak at 1180 cm^−1^ was assigned to the stretching vibrations of −S=O, which could be observed in the PNTS and PNS, but not in the PNT. This proved that SBMA units were successfully introduced into the PNTS and PNS hydrogels. As shown in Figure 2b,c, compared with the PNTS, the N−H stretching vibration peak of the a-PNTS shifted to 3273 cm^−1^, 3193 cm^−1^ and 3077 cm^−1^; the bending vibration peak of N−H shifted to 1540 cm^−1^; and the stretching vibration peak of C=O shifted to 1639 cm^−1^, indicating the presence of more hydrogen bonds inside the a-PNTS [37,38,39].

To analyze the chemical composition, X-ray photoelectron spectroscopy (XPS) was conducted to evaluate the a-PNTS and PNTS (Figure 2d). It was found that the contents of C, N, O and S in the PNTS and a-PNTS were calculated to be 64.9, 13.0, 21.0 and 1.0, and 64.0, 14.1, 20.8 and 1.1 at.%, respectively, indicating that the introduction of 1 wt.% ANFs did not affect the monomer polymerization (Table 2). The high-resolution C 1s spectra of the PNTS and a-PNTS could be deconvoluted into four component peaks, which could be attributed to C−C (284.6 eV), C−N (285.5 eV), C−O (286.2 eV) and C=O (287.6 eV), as shown in Table 3 and Figure 2e,f [22,40]. Whether ANFs were added or not, it could be found that the carbon environment in the hydrogel had no significant change. Due to the fact that the N element environment of the zwitterionic polymer PNTS and ANFs were mainly composed of amide N and −N^+^(CH_3_)_2_, the high-resolution N1s spectra of the PNTS and a-PNTS could be deconvoluted into two component peaks, which could be attributed to −N^+^(CH_3_)_2_ (402.1 eV) and O=C−N, with no significant changes in their contents (Figure 2g,h, Table 4) [22]. It is worth noting that the O=C−N peak binding energy of the PNTS was 399.3 eV, while the O=C−N peak binding energy of the a-PNTS was 399.7 eV. The O=C−N peak binding energy in the a-PNTS was closer to that of the PPTA fibers and PNAGA, which was attributed to the formation of more hydrogen bonds within the amide groups of the a-PNTS. The hydrogen bonds formed between nitrogen atoms in amide groups (ANFs and NAGA units), which led to a decrease in the electron cloud density of the N atoms, a decrease in the shielding effect and a core-level electron binding energy increase. Similar to the FTIR results, this proved that there were more hydrogen bonds between the amide groups in the a-PNTS, which resulted in more crosslinking points in the a-PNTS.

### 3.2. The Internal Microstructure

**Figure 3:**
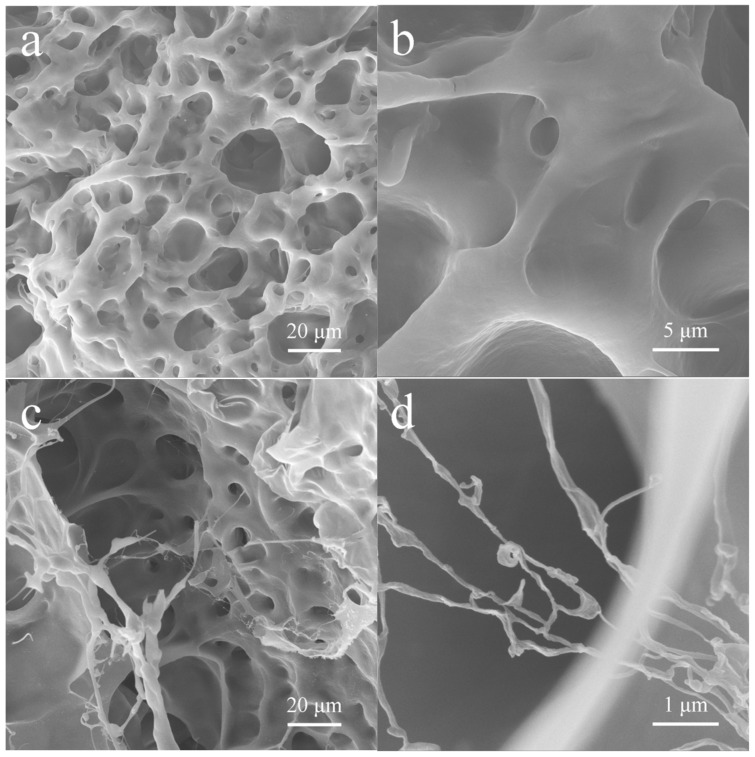
SEM images of internal structure of hydrogels: (**a**,**b**) PNTS and (**c**,**d**) a-PNTS.

The PNTS and a-PNTS hydrogels were immersed in water for 24 h to make them fully swelled, then cut and freeze-dried to obtain the aerogel formed by the swelled hydrogel. The internal microstructure was obtained by SEM. From Figure 3, it can be observed that the a-PNTS and PNTS exhibited a three-dimensional network, which was attributed to the chemical crosslinking formed during polymerization and the physical interactions between the polymer chains (hydrogen bonding and electrostatic adsorption). The PNTS exhibited a wide pore size distribution (5–100 μm) and wall thickness (2–30 μm). From the introduction of ANFs, many nanofibers with diameters of 40–60 nm could be observed in the three-dimensional network in the a-PNTS (Figure 3d). These nanofibers were extended from the wall and crosslinked between the walls. This may be attributed as follows: the NAGA, THMA and SBMA monomers adhered to the highly dispersed ANF surfaces through hydrogen bonding and then polymerized with the remaining monomers in the solution to form hydrogels. As the strength of the electrostatic adsorption force between the SBMA units and the hydrogen bond force between the THMA units were less than that of the hydrogen bond force between amides, the electrostatic adsorption force and the weak hydrogen bond (except the hydrogen bonds between amide groups) was partially broken during the swelling process of the hydrogels, while the hydrogen bonds between the amide groups in the ANFs and the NAGA did not break.

### 3.3. Mechanical Properties of Hydrogels

**Figure 4:**
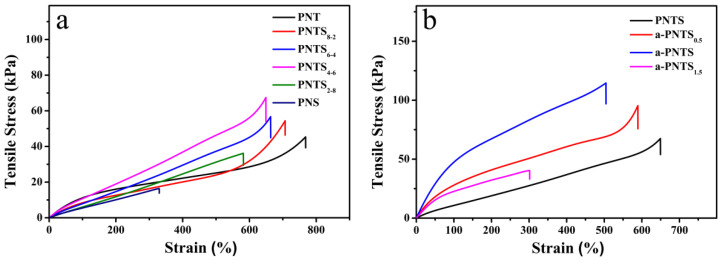
Tensile stress–strain curves of PNTS hydrogels with different (**a**) THMA/SBMA ratios and (**b**) ANF contents.

The mechanical properties of all the hydrogels were measured in tensile tests (Figure 4). First, the hydrogels with different mass ratios of THMA to SBMA were prepared under the condition that the content of the NAGA monomer was constant and the total contents of the THMA and SBMA monomers were fixed. They were named PNTS_x-y_, where x and y were the mass ratios of THMA and SBMA in the hydrogels, respectively. Tensile and compression tests were carried out on these hydrogels, and the results are shown in Figure 2. Without the addition of the SBMA monomer, the PNT gel showed good ductility, its elongation at break reached 768% and its tensile strength was 45 kPa. This was attributed as follows: with the introduction of SBMA and the reduction in the THMA content, the internal electrostatic adsorption of the hydrogels increased, the number of hydrogen bonds between sulfonic acid groups and other groups increased, the number of hydrogen bonds between hydroxyl groups and amide groups decreased, and the interaction of hydrogen bonds between amide groups increased, which improved the tensile strength. When the mass ratio of THMA/SBMA was less than 4 to 6, the tensile strength of the PNTS hydrogel decreased with the increase in the SBMA content. This was due to the long side chain of SBMA and the formation of too many hydrogen bonds between the sulfonic acid groups of SBMA units and NAGA units, which affected the formation of hydrogen bonds between the amide groups at the end of nearby NAGA units, which led to the reduction in the crosslinking degree, and ultimately, the tensile strength of the hydrogel.

Subsequently, tensile tests were carried out on the ANF-reinforced PNTS hydrogels with different dosages. The hydrogels were recorded as a-PNTS_x_, where x was the mass fraction of the added ANFs. The results are shown in Figure 4b. With the increase in the amount of ANFs, the tensile strength of the hydrogel showed a trend of first increasing and then decreasing. When 1 wt.% ANFs was added, the a-PNTS hydrogel showed a maximum tensile strength of 114 kPa, which was 1.7 times that of the pure PNTS hydrogel (67.5 kPa). It is worth noting that as the amount of ANFs added exceeded 1 wt.% and reached 1.5 wt.%, the tensile strength and break elongation of the a-PNST hydrogel decreased sharply. This was attributed to the following: ANFs rich in amide groups were used as “bridges” to form more crosslinking points in the a-PNTS hydrogels, which improved the tensile and compressive strengths of the hydrogel. When too many ANFs were added, they inevitably aggregated, which led to defects in the hydrogel, which greatly affected the strength and toughness of the hydrogel.

### 3.4. Swelling Behavior of Hydrogels

**Figure 5:**
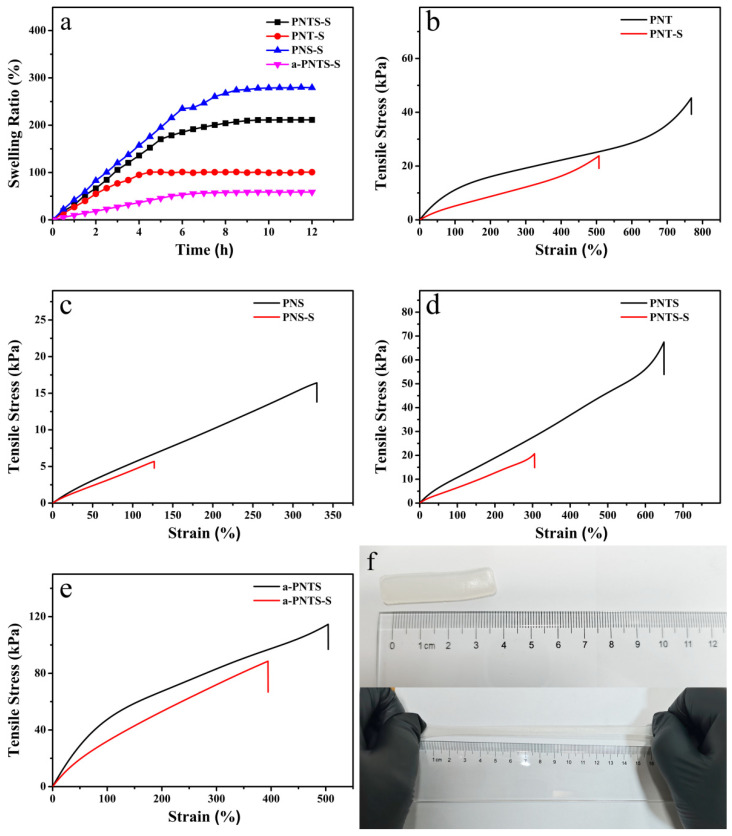
(**a**) Swelling ratios of PNS, PNT, PNTS and a-PNTS hydrogels. Tensile stress–strain curves of hydrogels before and after swelling: (**b**) PNT, (**c**) PNS, (**d**) PNTS and (**e**) a-PNTS. (**f**) Comparison photographs of a-PNTS-S hydrogels before and after stretching.

The PNT, PNS, PNTS and a-PNTS hydrogels were soaked in water, and their swelling ratios were recorded through their mass changes. The results are shown in Figure 5a. The fully swollen PNT, PNS, PNTS and a-PNTS hydrogels were named PNT-S, PNS-S, PNTS-S and a-PNTS-S. All samples were fully swollen within approximately 8 h. The swelling ratios of the PNT, PNS, PNTS and a-PNTS were 99.7%, 275.4%, 211.1% and 58.8%, respectively. This was attributed as follows: The extremely strong hydrophilicity of the side chains of SBMA units and the weak electrostatic adsorption force between their positive and negative groups, as well as the hydrogen bonds between the terminal hydroxyls of the THMA units and the terminal amide groups of the NAGA units, which resulted in the reduction in the number of strong hydrogen bonds (hydrogen bonds between amide groups) in the hydrogel. At the same time, the long side chains of SBMA also hindered the hydrogen bonding between the amide groups of nearby NAGA units, which resulted in less strong crosslinking inside the PNS and a higher swelling rate. From the results of the FTIR and XPS, due to the rich amide groups in the ANFs, the strong hydrogen bonds between amide groups in the PNTS polymer network were increased by the introduction of ANFs, which resulted in a higher crosslinking density of the polymer. The strong hydrogen bonds between amide groups did not break during the immersion in water, which led to a lower swelling ratio of the a-PNTS. Subsequently, for the PNT before and after swelling, the tensile properties of the PNS, PNTS and a-PNTS were tested, and the results are shown in Figure 5b–e. Due to the low swelling rate, the fully swollen a-PNTS hydrogel could still maintain a tensile strength of 88.5 kPa and an elongation at break of 394%.

**Figure 6:**
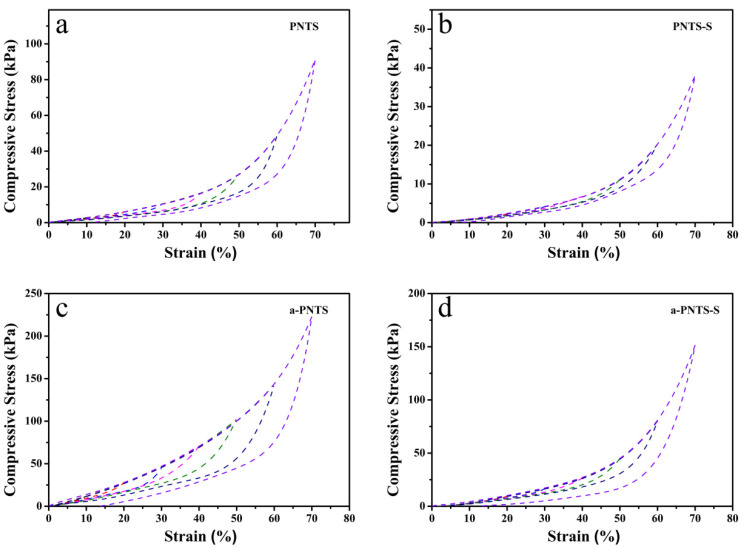
Compressive stress–strain curves of hydrogels: (**a**) PNTS, (**b**) PNTS-S, (**c**) a-PNTS and (**d**) a-PNTS-S.

The compression recovery performance of the PNTS and a-PNTS before and after swelling was tested (Figure 6). The maximum compressive recoverable strain of the PNTS and a-PNTS before and after expansion was 60%, and both the PNTS and a-PNTS after swelling exhibited small hysteresis loops. This was attributed to the large amount of electrostatic adsorption force between zwitterionic groups in the PNTS and a-PNTS before swelling, as well as the numerous weak hydrogen bonds that could repeatedly crosslink in the polymer network, which led to significant energy dissipation during the compression recovery. After swelling, the volume of a hydrogel becomes larger, the electrostatic adsorption force inside the hydrogel is weak, some weak hydrogen bonds break, the viscoelasticity is small and the energy dissipation is reduced [41]. The hysteresis loop of the a-PNTS after swelling was larger than that of the PNTS, which was attributed to the fact that the introduction of ANFs reduced the swelling of the a-PNTS hydrogels and retained the electrostatic adsorption force generated between some internal zwitterionic groups.

### 3.5. The Strain-Sensing Performance

**Figure 7:**
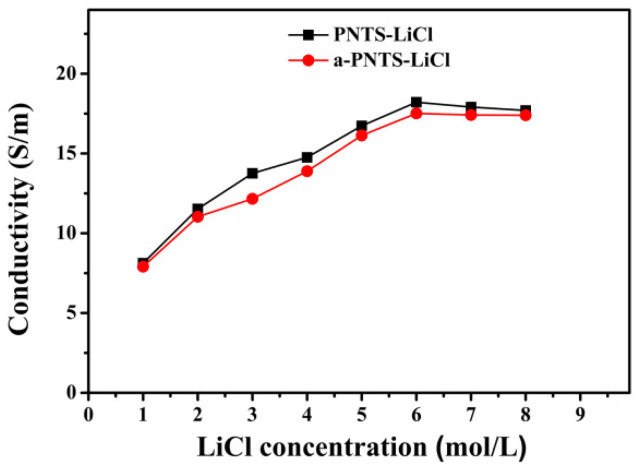
Ionic conductivity of PNTS and PNTS hydrogels in LiCl with different concentrations.

The PNTS and a-PNTS hydrogels were soaked in lithium chloride solutions with different concentrations for 24 h, and then their ionic conductivities were measured (Figure 7). The ionic conductivity of the PNTS and a-PNTS hydrogels increased with the increase in the LiCl solution concentration, and reached the maximum when the LiCl concentration was 6 mol L^−1^, which indicates that the combination of sulfonic groups, amino groups, carbonyl groups and hydroxyl groups of the hydrogel with Li^+^ and the combination of −N^+^(CH)_2_ and Cl^−1^ produced saturation. At this time, the ionic conductivities of the PNTS and a-PNTS were 18.2 S m^−1^ and 17.5 S m^−1^, respectively. It was found that the ionic conductivity of the a-PNTS hydrogel was slightly lower than that of the PNTS hydrogel, which indicates that the introduction of ANFs built more strong hydrogen bonds inside the hydrogel and reduced the combination between some functional groups and LiCl.

**Figure 8:**
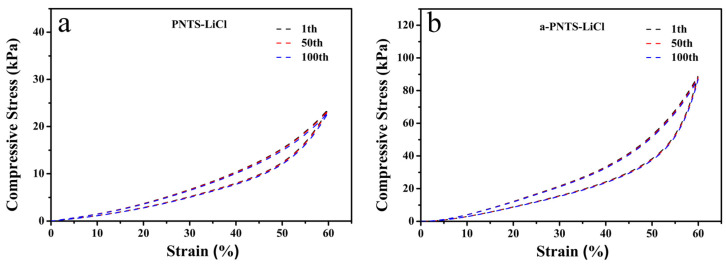
Compressive cyclic stress–strain curves of hydrogels with different cycles: (**a**) PNTS-LiCl and (**b**) a-PNTS-LiCl.

The PNTS and a-PNTS hydrogels soaked in 6 M LiCl were named PNTS-LiCl and a-PNTS-LiCl, respectively, and their compression recovery stabilities under 60% compression strain were tested (Figure 8). It was found that after 100 cycles of compression, the compression strengths of the PNTS-LiCl and a-PNTS-LiCl hydrogels were almost undamaged, which means that they have the potential to be used as pressure sensors.

**Figure 9:**
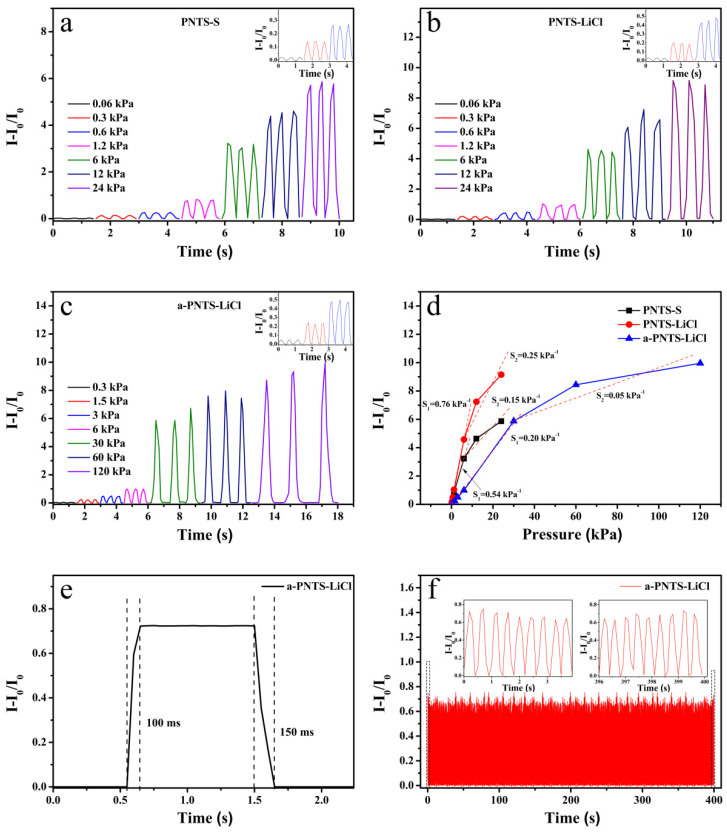
Relative current changes of the sensor under compressive pressure: (**a**) PNTS-S, (**b**) PNTS-LiCl and (**c**) a-PNTS-LiCl. (**d**) Sensitivities of the PNTS-S, PNTS-LiCl and a-PNTS-LiCl sensors. (**e**) Response and recovery times of the PNTS-LiCl sensor under a pressure of 6 kPa. (**f**) The compressive cyclic stability test of the a-PNTS-LiCl sensor under 6 kPa for 1000 cycles.

Figure 9 shows the current responses of the PNTS-S and PNTS-LiCl sensors at different pressures, demonstrating typical piezoresistive behavior [42]. It can be seen from this figure that the PNTS-S and PNTS-LiCl sensors exhibited higher sensitivity at low pressures, which could be attributed to the large deformation that could be generated in the low-pressure region, which led to a rapid shortening of the ion channel and resulted in a significant decrease in the resistance and an increase in the current. With the increase in pressure, the gradual densification of the 3D network structure led to little change in the hydrogel deformation and limited ion migration, so the current change was small and the pressure sensitivity was reduced. From Figure 9c,d, it can be seen that the sensitivity and current response of the PNTS-LiCl was higher than the PNTS-S under the same pressure. This was attributed to the introduction of LiCl, which constructed more ion transport channels in the PNTS-LiCl and resulted in greater changes in the current with changes in the pressure.

By introducing ANFs, the a-PNTS-LiCl sensor had a higher compression strength, and compared with the PNTS-LiCl sensor, the a-PNTS-LiCl sensor had a regular current response over a larger pressure range (0.3–120 kPa) (Figure 9d). The response and recovery times of the a-PNTS were 100 ms and 150 ms, respectively, showing a rapid response and recovery to mild external stimuli (Figure 9e). This was attributed to the fact that the hydrogel prepared showed low viscoelasticity and a small hysteresis loop after immersion in 6 M lithium chloride, which indicates that less energy was dissipated during the compression recovery and resulted in a rapid response and recovery of the hydrogel. The current response of the a-PNTS under 1000 compressions was tested to assess its stability, and its amplitude and waveform remained consistent (Figure 9f), making it suitable for long-term practical applications. The sensing performances of the PNTS-LiCl and a-PNTS-LiCl pressure sensors exceeded those of some reported advanced pressure sensors (details can be found in Table 5).

**Figure 10:**
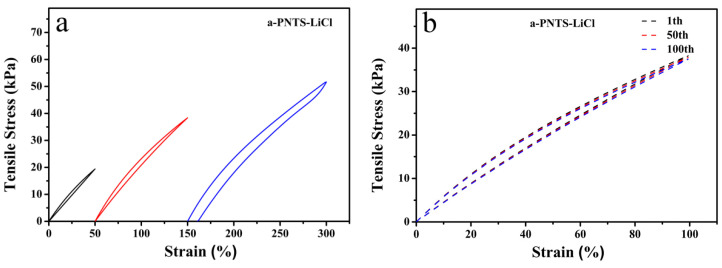

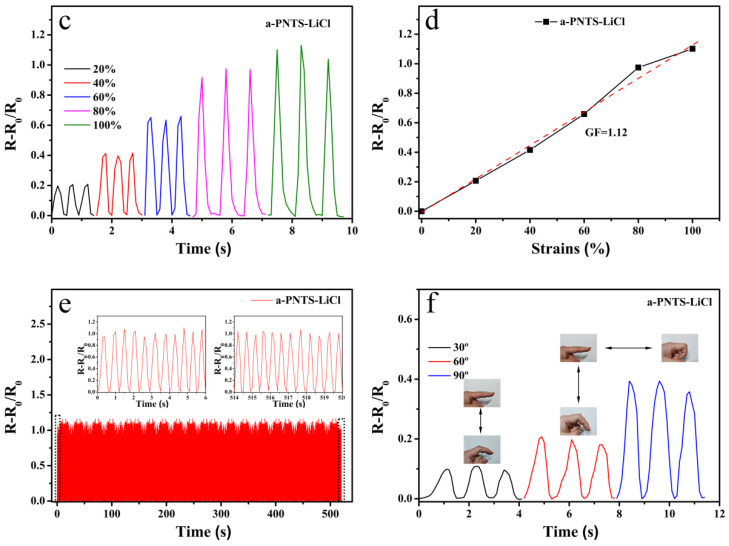
(**a**) Tensile stress–strain curves of the a-PNTS-LiCl hydrogel. (**b**) Tensile cyclic strength–strain curves of the a-PNTS-LiCl hydrogel. (**c**) Relative current change of the a-PNTS-LiCl sensor under different strains in the range from 0 to 100%. (**d**) GF of the PNTS-LiCl sensor. (**e**) The tensile cyclic stability test of the a-PNTS-LiCl sensor under 100% strain for 1000 cycles. (**f**) Current–time curve with different finger-bending angles.

Due to the excellent tensile properties of the a-PNTS-LiCl, we found that its tensile strength could be restored to 100% tensile strain and remained stable after 100 tensile cycles (Figure 10a,b). This means that it can also serve as a stretch sensor. The current responses of the a-PNTS under different tensile strains were tested. As shown in Figure 10, the a-PNTS-LiCl sensor exhibited regular current changes under different tensile strains, with a gauge factor (GF) of 1.12. At the same time, it was fixed at the finger joint to identify whether it could detect human movement. It can be observed that the current response increased regularly as the finger joint bent back and forth at angles of 30°, 60° and 90°. This indicates that it has great potential for application in wearable devices for detecting human motion.

## 4. Conclusions and Outlook

In conclusion, a new zwitterionic polymer hydrogel (PNTS) was prepared by one-step radical polymerization of NAGA, THMA and SBMA monomers. The prepared hydrogel had good compressive and tensile strengths. When ANFs rich in amide groups were introduced into the zwitterionic polymer hydrogel (a-PNTS), a large number of hydrogen bonds were formed between the ANFs and the NAGA/THMA/SBMA units, which greatly increased the internal crosslinking degree of the hydrogel. The tensile strength of a-PNTS hydrogel was 1.7 times higher than that of the PNTS hydrogel without ANFs. Due to the higher crosslinking degree within the a-PNTS, the swelling rate of the a-PNTS hydrogel was 58.8%, which was much lower than the swelling rate of the PNTS hydrogel (211.2%). The prepared PNTS and a-PNTS hydrogels exhibited excellent compression and tensile recoverability. When assembled into sensors, they exhibited excellent stability and fast responses and could effectively detect the activity of human joints. The zwitterionic hydrogel-based strain sensor with excellent performance prepared by this method only required mixing the ANFs, monomer and initiator (APS) in water, then injecting them into a specific mold for heating to polymerization. Different shapes can be customized according to actual applications and the forming time is short, which exhibits enormous potential practical value in the application of multifunctional flexible sensors with large-scale production.

## Figures and Tables

**Table 1:** The main compositions of prepared hydrogels.

Sample	NAGA (mg)	THMA (mg)	SBMA (mg)	ANFs (mg)	DI Water (mL)
PNS	400	0	200	0	2
PNT	400	200	0	0	2
PNTS	400	80	120	0	2
PNTS_2-8_	400	40	160	0	2
PNTS_6-4_	400	120	80	0	2
PNTS_8-2_	400	160	40	0	2
a-PNTS-0.5	400	80	120	3	2
a-PNTS	400	80	120	6	2
a-PNTS-1.5	400	80	120	9	2

**Table 2:** The chemical composition of a-PNTS and PNTS from XPS analysis.

Sample	C (at.%)	N (at.%)	O (at.%)	S (at.%)
PNTS	64.9	13.0	21.1	1.0
a-PNTS	64.0	14.1	20.8	1.1

**Table 3:** Decomposed C 1s energy state of a-PNTS and PNTS from XPS analysis.

Sample	C−C (284. 6 eV)	C−N (285.5 eV)	C−O (286.2 eV)	C=O (287.6 eV)
PNTS	49.5	8.5	10.5	31.5
a-PNTS	45.8	9.7	10.2	34.4

**Table 4:** Decomposed N 1s energy state of a-PNTS and PNTS from XPS analysis.

Sample	C−N	−N^+^ (CH_3_)_2_ (402.1 eV)
PNTS	92.7	7.3
a-PNTS	92.1	7.9

**Table 5:** Comparison of sensitivity, detection range, detection limit and response time of reported pressure sensing materials.

Material	Sensitivity (Detection Rage)	Detection Limit	Response Time, Recovery Time	Ref
PAAm/AKP/Alg-Zn^2+^	0.35 kPa^−1^ (<5 kPa) 0.05 kPa^−1^ (5–50 kPa)	-	200 ms, 200 ms	[43]
P(AA-APA)-Fe^3+^	0.015 kPa^−1^ (<20 kPa) 0.0072 kPa^−1^ (20–70 kPa) 0.0022 kPa^−1^ (70–150 kPa)	1 kPa	180 ms, 170 ms	[44]
APS/AgNW	0.30 kPa^−1^ (<1 kPa) 0.033 kPa^−1^ (1–14 kPa) 2.27 MPa^−1^ (14–64 kPa) 0.32 MPa^−1^ (64–250 kPa)	-	271 ms, 296 ms	[45]
PMAA@MXene	5.15 MPa^−1^ (<0.09 MPa) 0.09 MPa^−1^ (0.09–1.82 MPa)	-	0.75 s, -	[46]
NaCl-TA-PAM	0.95 kPa^−1^ (<0.448 kPa) 0.17 kPa^−1^ (0.638–1.641 kPa)	16 Pa	20 ms, 40 ms	[47]
SFRHs	0.038 kPa^−1^ (<16.5 kPa) 0.008 kPa^−1^ (16.5–28 kPa)	1 kPa	-	[48]
CMGG/PAA/LS/Al^3+^	0.104 kPa^−1^ (<2.5 kPa) 0.015 kPa^−1^ (16.5–24 kPa)	-	200 ms, 200 ms	[49]
PNTS-LiCl	0.76 kPa^−1^ (<6 kPa) 0.25 kPa^−1^ (6–24 kPa)	60 Pa	100 ms, 150 ms	This work
a-PNTS-LiCl	0.20 kPa^−1^ (<30 kPa) 0.05 kPa^−1^ (30–120 kPa)	300 Pa	100 ms, 150 ms	This work

## Data Availability

The original contributions presented in this study are included in the article. Further inquiries can be directed to the corresponding author.
